# Construction of Transgenic *Plasmodium berghei* as a Model for Evaluation of Blood-Stage Vaccine Candidate of *Plasmodium falciparum* Chimeric Protein 2.9

**DOI:** 10.1371/journal.pone.0006894

**Published:** 2009-09-03

**Authors:** Yi Cao, Dongmei Zhang, Weiqing Pan

**Affiliations:** Department of Pathogen Biology, Second Military Medical University, Shanghai, China; Singapore Immunology Network, Singapore

## Abstract

**Background:**

The function of the 19 kDa C-terminal region of the merozoite surface protein 1 (MSP1-19) expressed by *Plasmodium* has been demonstrated to be conserved across distantly related *Plasmodium* species. The green fluorescent protein (GFP) is a reporter protein that has been widely used because it can be easily detected in living organisms by fluorescence microscopy and flow cytometry.

**Methodology and Results:**

In this study, we used gene targeting to generate transgenic *P. berghei* (Pb) parasites (designated as PfMSP1-19Pb) that express the MSP1-19 of *P. falciparum* (Pf) and the GFP reporter protein simultaneously. The replacement of the PbMSP1-19 locus by PfMSP1-19 was verified by PCR and Southern analysis. The expression of the chimeric PbfMSP-1 and the GFP was verified by Western blot and fluorescence microscopy, respectively. Moreover, GFP-expressing transgenic parasites in blood stages can be readily differentiated from other blood cells using flow cytometry. A comparion of growth rates between wild-type and the PfMSP1-19Pb transgenic parasite indicated that the replacement of the MSP1-19 region and the expression of the GFP protein were not deleterious to the transgenic parasites. We used this transgenic mouse parasite as a murine model to evaluate the protective efficacy in vivo of specific IgG elicited by a PfCP-2.9 malaria vaccine that contains the PfMSP1-19. The BALB/c mice passively transferred with purified rabbit IgG to the PfCP-2.9 survived a lethal challenge of the PfMSP1-19Pb transgenic murine parasites, but not the wild-type *P. berghei* whereas the control mice passively transferred with purified IgG obtained from adjuvant only-immunized rabbits were vulnerable to both transgenic and wild-type infections.

**Conclusions:**

We generated a transgenic *P. berghei* line that expresses PfMSP1-19 and the GFP reporter gene simultaneously. The availability of this parasite line provides a murine model to evaluate the protective efficacy in vivo of anti-MSP1-19 antibodies, including, potentially, those elicited by the PfCP-2.9 malaria vaccine in human volunteers.

## Introduction

There is an urgent need for the development of a malaria vaccine to control the tropical disease because of the emergence and rapid spread of drug-resistant parasites and insecticide-resistant mosquitoes [1]. The 185–215 kDa merozoite surface protein 1 of *Plasmodium falciparum* (PfMSP1) is a leading anti-blood stage malaria vaccine candidate [2], [3]. The PfMSP1 undergoes proteolytic processing during merozoite maturation resulting in four major fragments of 83, 30, 38 and 42 kDa [4]. Before erythrocyte invasion, the 42-kDa fragment undergoes a secondary proteolytic cleavage, leaving the C-terminal 19-kDa fragment (MSP1-19) associated with merozoites in newly invaded erythrocytes [5]. Antibodies that are specific for MSP1-19 comprise a large component of the inhibitory activities observed in naturally exposed individuals [6], [7]. Several vaccination studies using MSP1-19 in both mice and monkeys have shown partial protection against parasite challenge [8], [9]. In addition to PfMSP1, the apical membrane antigen of *Plasmodium falciparum* (PfAMA-1) is another attractive vaccine candidate against blood-stage parasite [2], [10]–[12]. The most C-terminal of the disulphide-bonded domains in AMA-1 (AMA-1(III)) was demonstrated to be the target of inhibitory antibodies isolated from humans in malaria-endemic regions [13].We have constructed a *Plasmodium falciparum* chimeric protein that consists of AMA-1(III) and MSP1-19 (designated as PfCP-2.9) [14], [15]. The sera from vaccinated rabbits and rhesus monkeys with PfCP-2.9 formulated by Montanide ISA 720 almost completely inhibited *in vitro* growth of *P. falciparum* FCC1/HN and 3D7 lines. The PfCP-2.9 vaccine candidate is being tested in clinical trials [16], [17].

Malaria vaccine development requires appropriate experimental animal models for the evaluation of the efficacy of a vaccine in vivo. Although trials in Aotus monkeys have provided important information on the protective efficacy of malaria vaccine candidates, the use of this monkey species was limited [18]. Although growth inhibition assays could provide information about the inhibitory function of antibodies, these provide only in vitro assessment and do not represent the true efficacy of immune sera in vivo [19], [20]. Therefore, alternative models for the in vivo evaluation of vaccine efficacy are urgently needed.

It was recently reported that transgenic murine malaria parasites that express human malaria genes were generated for the assessment of vaccine-inducing antibodies [21]–[23]. Although the role of the PfMSP1 gene is essential for development of the blood stage of the parasite, allelic replacement experiments have shown that the function of the MSP1-19 fragment is highly conserved across distantly related *Plasmodium* species [24]. The green fluorescent protein (GFP) is a well-established reporter protein that has been exploited in a large variety of cell-type analysis systems [25]. Stable episomal or integrated expression of GFP has been reported in *Plasmodium* for the examination of gene expression, protein localization and biological processes [26], [27]. In this study, we utilized gene targeting to develop transgenic *P. berghei* parasites that expressed *P. falciparum* MSP1-19 as well as GFP. This transgenic parasite line can be readily detected in all blood stages using fluorescent microscope and flow cytometry. The growth characteristics of the fluorescent transgenic parasites in the blood stage were similar to wild type *P. berghei.* We employed transgenic rodent parasites as an *in vivo* murine model to further assess the protective efficacy of anti-MSP1-19 antibodies elicited by PfCP-2.9.

## Materials and Methods

### Parasites and experimental animals

The ANKA strain of *P. berghei* used in this study is an uncloned line that was obtained from the National Institute for Parasitic Diseases of the Chinese Center for Disease Control and Prevention. The *P. berghei* ANKA parasites were maintained by mechanical passage in mice.

Inbred BALB/c mice, outbred KM mice and New Zealand White rabbits were purchased from the Shanghai Laboratory Animal Center of the Chinese Academy of Sciences. All of the mice were housed in specific pathogen free (SPF) standards while the rabbits were housed in a conventional animal facility. All of the procedures performed on animals within this study were conducted in accordance with the guidelines of the Committee on Animals of the Second Military Medical University.

### Plasmid construction

The *P. berghei* transfection vector pPyrFlu which contains the GFP gene, was generously provided by Dr. Robert Ménard (Institut Pasteur, Paris, France) [28]. To create the PbfMSP1 fusion gene, the 1,423-base pair (bp) *P. berghei* MSP1 targeting sequence and the 330-bp PfMSP1-19 fragment were PCR amplified from a *P. falciparum* FCC1/HN isolate and *P. berghei* ANKA genomic DNA, respectively, using primers PbF (5′-CGCGAGCTCTTAACAAAAGAAGAGAAGC-3′) and PbR (5′-GCATACATGCTTAGGGTCTATACCTAATAAATC-3′), and PfF (5′-GGTATAGACCCTAAGCATGTATGCGTAAAAAAACAATGTCCAG-3′) and PfR (5′-CGCGTCGACTTAAATGAAACTGTATAATATTAAC-3′). The two PCR products were fused in frame by using the primers PbF and PfR and resulted in PbfMSP1. The 860-bp PfHSP86 3′ untranslated region (UTR) was amplified from plasmid pHC1 (a gift from the Malaria Research and Reference Reagent Resource Center) using the primers HSPF (5′-CGGCTCGAGTTATATAATATATTTATGTAC-3′) and HSPR (5′-CGGGGATCCTATTTGATGAATTAACTACAC-3′) and ligated in the correct orientation to PbfMSP1 to create a 2.6 kb fragment that was cloned into the *Sac*I/*Bam*HI sites of pPyrFlu. The other 0.5 kb targeting sequence of PbMSP-1 3′UTR was also amplified from *P. berghei* ANKA genomic DNA using the primers Pb3′F (5′-GGCGGGCCCATAAATTATTGAAATATTTGTTG-3′) and Pb3′R (5′-CGCGGTACCTATACAAAACATATACA-3′) and then cloned into the *Apa*I/*Kpn*I sites of pPyrFlu. The resulting vector containing PyrFlu/PbfMSP-1/ PbM3′ was linearized with *Kpn*I and *Sac*I before transfection.

### Transfection, cloning and genotype analysis of transgenic parasites

**Figure 1:**
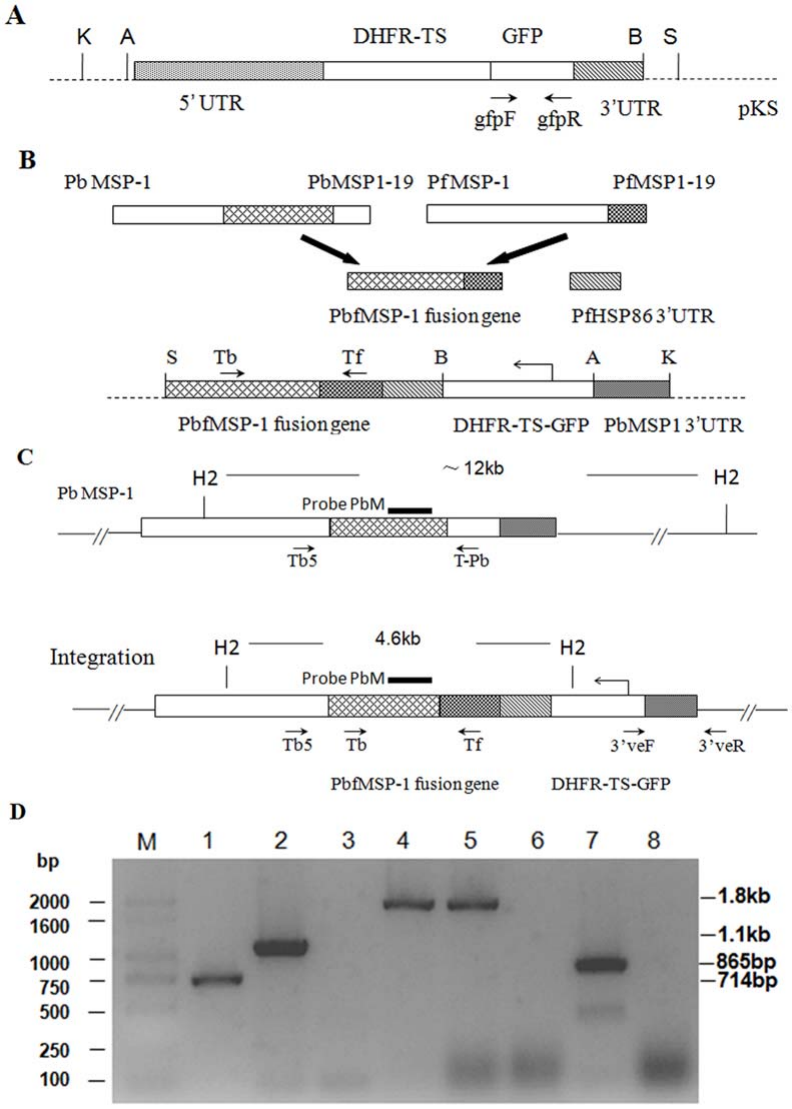
Generation of mutant *P. berghei* parasites and PCR analysis. Organizational maps of plasmid PyrFlu (A), the construction strategy of recombinant vector PyrFlu/PbfMSP-1/ PbM3′ (B), the gene map following the homologous integration of plasmid PyrFlu/PbfMSP-1/ PbM3′ at the MSP1 locus (C). Genomic DNA of PfMSP1-19Pb_8.7_ clone (lanes 1–4 and 7) and wild-type *P. berghei* ANKA (lanes 5–6 and 8) were used as template; Test primers are indicated by arrows. lane 1: amplification of *gfp* with the primers *gfp*F/*gfp*R; lane 2: verification of transfection using primers Tb/Tf; lanes 3 and 5: verification of the wild-type PbMSP-1 locus with the primers Tb5/T-Pb; lanes 4 and 6: verification of the predicted 5′ integration into the PbMSP-1 locus with the primers Tb5/Tf; lanes 7 and 8: verification of the predicted 3′ integration into the PbMSP-1 locus with the primers 3′veF/3′veR; M: base pair ladder (D). Probe PbM for Southern analysis was shown as black bar. The expected sizes of fragments resulting from digestion with HincII are shown. S (*Sac*I), B (*BamH*I), A (*Apa*I), K (*Kpn*I) and H2(*HincII*).

Transfection of the blood stages of the *P. berghei* ANKA strain and the selection of transfected parasites was performed as described previously [29]. Individual clones were obtained by injecting 1 infected erythrocyte per mouse intravenously. Briefly, the blood cells were collected from an infected mouse and diluted with complete medium to generate the cell suspension. Then, we determined the erythrocyte density of the cell suspension using a blood cell-counter. The number of infected cells in the suspension was calculated by the erythrocyte density and the parasitemia determined on Giemsa-stained blood smears. The cell suspension was further diluted with complete culture medium to 1 infected RBC per 100 µl. Thus, 100 µl of this suspension was injected into mice through the tail vein. For analysis of the genotype of post-transfection parasite clones, genomic DNA was isolated from the blood of asynchronous infected mice using a TIANamp Blood Genomic DNA Kit (Tiangen Biotech, Beijing, China) and analyzed by PCR and Southern blot. The correct integration was determined by PCR using the test primers as described below. The presence of the GFP reporter gene was confirmed by PCR using the primers *gfp*F (5′-ATGAGTAAAGGAGAAGAACTTTTC-3′) and *gfp*R (5′-TTATTTGTATAG TTCATCCATGC-3′). The presence of the transfection vector in respective clones was detected by PCR using the primers Tb (5′ –CATCGACACCAGAAGAAGTAGCAAG-3′) and Tf (5′-CCGTTGCTACCTGAATCTTCTTCG-3′), which amplified PbfMSP1 fused from 1.4 kb of the *P. berghei* MSP1 targeting sequence and 330 bp of the PfMSP1-19 fragment. To verify the 5′ integration fidelity at the *P. berghei* MSP1 gene, the primers Tb5 (5′-GAAATCGCACACTTAAAGGAATTATCAGAG-3′), specific for a sequence upstream of the 1.4 Kb *P. berghei* MSP1 targeting sequence, and Tf were used. As a control, the primers Tb5 and T-Pb (5′-GAATTTTCCCTATTTTCCGCAGTT-3′), specific to *P. berghei* MSP1-19, were used in the PCR to identify the wild-type parasites. To verify the 3′ integration fidelity, the primer 3′veF (5′ –TACACAAACATACAAAAATAAAC-3′), specific to *P. berghei* DT 5′UTR, and the primer 3′veR (5′ –ATAATACAACAAAATGGATA-3′), specific to a sequence downstream of the *P. berghei* MSP1 3′UTR targeting sequence, were used. Schematic representations of all of the primers are shown figures 1A, B and C.

We also performed a Southern blot experiment to further confirm the integration using a 430-bp probe prepared by PCR amplification of the fragment derived from the transfection vector PyrFlu/PbfMSP-1/PbM3′ using the primers PbMF (5′-TAAAATATGCTGCTAAAG-3′) and PbMR (5′-GATTCGCCTTCTCCTACC-3′). Individual HincII-digested genomic DNA fragments of wild-type and transgenic *P. berghei* parasites were hybridized to the probe by standard procedures [30] following the manufacturer's instructions of the North2South® direct HRP labeling and detection kit (Pierce, Rockford, IL, USA). Schematic representations of the probe and digestion fragments are shown the figures 1C.

In order to test the passage stability, 10^6^ transgenic *P. berghei* parasites were inoculated intraperitoneally (i.p.) into 5 naïve KM mice without pyrimethamine pressure. Each week, 50 µl of blood taken from infected mice was diluted in 500 µl PBS and passaged into 5 new KM mice. This was repeated 5 times. The infected blood collected from each passage was used for the extraction of genomic DNA. Ten micrograms of genomic DNA was digested by HincII overnight and subjected to Southern analysis according to the above-mentioned protocol. The genomic DNA from wild type *P. berghei* was used as a control.

### GFP detection and flow cytometry analysis of blood stages

Blood samples were collected from the tails of mice infected with the transgenic clones. Thin smears was prepared on the slides and air-dried completely at room temperature. We did not take any fixation procedure to the blood smears before fluorescence detection. GFP fluorescence in the different blood stages was visualized and recorded using the GFP filter settings of a Leica TCSSP2 laser scanning confocal microscope.

The GFP fluorescence intensity of transgenic parasites in blood stages was analyzed by flow cytometry using a FACSCalibur (Becton Dickinson, CA, USA). Excitation of cells was performed with an argon ion laser at a wavelength of 488 nm and the emission of the green fluorescence was detected using a band pass filter of 530/30 nm. To determine the consistency between the parasitemia (the percentage of infected erythrocytes) automated counted by flow cytometry and the parasitemia manually counted in Giemsa stained blood films, 5 naïve female BALB/c mice were intraperitoneally inoculated 2×10^5^ transgenic *P. berghei* parasites. Tail blood was collected from each mouse (10 µl per sample) in heparin (1 U/ul) daily, diluted in 2–3 ml of PBS, and then analyzed using the FACSCalibur. In these samples, erythrocytes were selected on size for analysis by gating on forward/side-light scatter. By gating the uninfected erythrocytes and the GFP-positive infected erythrocytes, parasitemia was determined by flow cytometry in the infected mice with parasitemia levels between 0.1 and 12%. Giemsa-stained blood films were also prepared and counted daily. The data were analyzed with SPSS15.0.

### Western blot analysis

The *P. falciparum* and *P. berghei* schizont- or merozoite-infected erythrocytes were harvested, washed three times with phosphate buffered saline (PBS, pH 7.4) and pelleted by centrifugation (1,500 rpm for 8 min at room temperature). The pellets were lysed in an equal volume of 0.15% saponin in PBS for 30 min at 37°C and the released parasites were pelleted, washed 3 times with ice-cold PBS (10,000 rpm for 10 min at 4°C). The parasite pellet was resuspended in 1 mM phenylmethylsulfonyl fluoride (PMSF) and 100 mM EDTA, repeatedly freezed and thawed using liquid nitrogen, incubated in Solution Buffer (50 mM Tris-HCl, 10 mM EDTA, 1 mM PMSF, 1% Triton X-100) on ice for 1 h and then centrifuged (10,000 rpm for10 min at 4°C) to remove insoluble materials. Lysate supernatants were separated using nonreducing 8% and 12% sodium dodecyl sulfate polyacrylamide gel electrophoresis (SDS-PAGE) gels and transferred onto nitrocellulose (NC) membranes. The membranes were blocked with 3% non-fat dry milk/PBS, pH 7.4, and probed at 37°C for 1 h with either affinity-purified anti-PfMSP1-19 rabbit polyclonal antibodies [14] or anti-PbCP-2.9 (recombinant PbCP-2.9 also contains the PbMSP1-19 antigen) mouse sera [31], both diluted 1∶200 in 3% non-fat dry milk/PBS, pH 7.4. Alkaline phosphatase-conjugated goat anti-rabbit or goat anti-mouse secondary antibodies were diluted 1∶500 in 3% non-fat dry milk/PBS and added to the membranes at 37°C for 1 h. At every step, the membranes were washed three times with PBS containing 0.05% Tween 20, pH 7.4. Bands were visualized using NBT (Nitro-Blue Tetrazolium Chloride) and BCIP (5-Bromo-4-Chloro-3′-Indolyphosphate p-Toluidine Salt) as chromogenic substrates.

### Growth rates of Transgenic P. berghei parasites

To compare growth rates, two groups of female BALB/c mice, 6–8 weeks of age (5 mice/group), were injected i.p. with 10^4^ transgenic or wild-type *P. berghei*. Parasitemia was assessed daily in Giemsa-stained thin smears prepared from tail blood of each mouse. Parasitized erythrocytes were counted and the data were expressed as the mean±SD of observations and statistical differences were analyzed using the Mann-Whitney test.

### Immunization with PfCP-2.9 and IgG purification

New Zealand White rabbits (n = 3) were subcutaneously (s.c.) immunized with 100 µg of PfCP-2.9 antigen emulsified with Montanide ISA 720 adjuvant (Seppic Inc., Paris, France) while control rabbits (n = 3) received only PBS emulsified with Montanide ISA 720. All rabbits received three immunizations at 2-week intervals with the same dose of antigen and adjuvant. Bleeding was carried out on days 0 (pre-immune), 21 and 42, and the sera were used for ELISA analysis and for IgG purification.

Total IgG was purified from rabbit sera using protein G columns (Pierce Inc., Rockford, IL, USA). Briefly, rabbit sera was diluted at least 1∶2 with Binding Buffer (BB) (20 mM Tris-HCl, pH 7.4) and loaded onto a protein G-Sepharose column. The column was washed with 10-column volumes of BB, and the bound IgG was eluted with 0.2 M glycine-HCl (pH 2.5). The pH was immediately adjusted to 7–8 with 1 M Tris-HCl (pH 9.0). The eluted fractions were dialyzed against RPMI 1640 (Invitrogen, Carlsbad, CA, USA) and concentrated with centrifugal filter devices (Millipore, Billerica, MA USA) to a concentration above 20 mg/ml. Purified IgG was sterilized by filtration through a 0.22-µm filter (Millipore). The integrity and purity of the antibodies was verified on SDS-PAGE gels.

### Passive transfer of IgG and parasite challenge

To test the protective efficacy of rabbit antibodies generated against PfCP-2.9, purified IgG was passively transferred intravenously (i.v.) into two groups of BALB/c mice (6–8 weeks old; 4 mice/group) given either 0.5 or 1.0 mg per dose at three times (on days −1, 0 and +1 with respect to the parasite challenge). Thus, the total doses of purified IgG for individual mouse were 1.5 and 3.0 mg, respectively. Control mice received purified IgG at the same doses from adjuvant-only immunized rabbits. Transgenic *P. berghei* were used for the parasite challenge. Parasitized erythrocytes (500/mouse) were injected i.v. 1 h after the IgG administration on day 0. A similar set of four groups of mice that were immunized in the same way was challenged with wild-type *P. berghei*. Parasitemia was assessed daily in Giemsa-stained blood smears prepared from each mouse. The time of death was observed for all groups, and experiments were carried out twice.

## Results

### Construction of the transfection vector

Since integration into *Plasmodium spp.* genomes usually occurs via homologous recombination, the two endogenous *P. berghei* fragments, *msp*-1 (1.4 kb) and the 3′UTR of PbMSP1 (0.5 kb), were amplified from genomic DNA of *P. berghei* ANKA strain as targeting sequences. The 3′UTR of PbMSP-1 was cloned into the *Kpn*I/*Apa*I restriction sites upstream of the DHFR-TS/GFP*mut2* fusion gene in the PyrFlu plasmid while the 1.4 kb *P. berghei msp*-1 fragment was fused in frame with the 0.33 kb *msp*1-19 region of *P. falciparum* and ligated to the PfHSP86 3′UTR at the 3′ end of PbfMSP-1 as a termination sequence. The PbfMSP-1/HSP86 3′ fragment was cloned into the *Sac*I/*BamH*I restriction sites downstream of the DHFR-TS/GFP*mut2* fusion gene, creating the PyrFlu/PbfMSP-1/PbM3′ vector. The complete sequences of all the amplified fragments were verified by sequencing, and the transfection vector was confirmed by PCR and enzyme digestion. After linearization at the *Kpn*I/*Sac*I restriction sites, the vector could be integrated into the MSP1 locus of *P. berghei* by a double crossover event.

### Genotype analysis of post-transfection parasites

The linear form of the transfection vector was introduced into the blood stages of *P. berghei* by electroporation, and parasites were selected using pyrimethamine. When green fluorescence was observed in pyrimethamine-resistant parasites, cloning was performed by limiting dilution. Genomic DNA of each clone was extracted, and the correct integration was firstly verified by PCR using the test primers. Since the vector could integrate into the MSP1 gene locus of *P. berghei*, the PfMSP1-19 fragment derived from *P. falciparum* replaced the corresponding endogenous sequence of *P. berghei*. The 0.7 kb *gfp* fragment and 1.1 kb fragment of the PbfMSP-1 fusion gene were amplified by the *gfp*F/*gfp*R and Tb/Tf primers, respectively, to verify the presence of the transfection vector in the parasite genome. The 1.8 kb fragment amplified by the Tb5/Tf primers demonstrated the correct 5′ integration into the *msp*1 gene locus of *P. berghei*, and the amplification of the1.8 kb fragment was done using the Tb5/T-Pb primers as controls for detecting the wild-type *P. berghei msp*1 gene. The 3′ integration fidelity was confirmed by PCR amplification of the 0.86 kb fragment using the primer pair 3′veF/3′veR.

During the first cloning phase eight (*i.e.* 1, 2, 7, 8, 10, 14, 15 and 19) of the pyrimethamine-resistant lines showed green fluorescence, but PCR amplification of these eight lines using the Tb5/Tf and Tb5/T-Pb primers resulted in 1.8 kb bands, suggesting that a mixed population containing wild-type and transgenic parasites was present. We chose parasite line 8 as the initial population for the second cloning, and obtained 4 clones (defined as 8.1, 8.4, 8.7 and 8.11) that had the correct vector integration as confirmed by PCR analysis (figure 1D).

**Figure 2:**
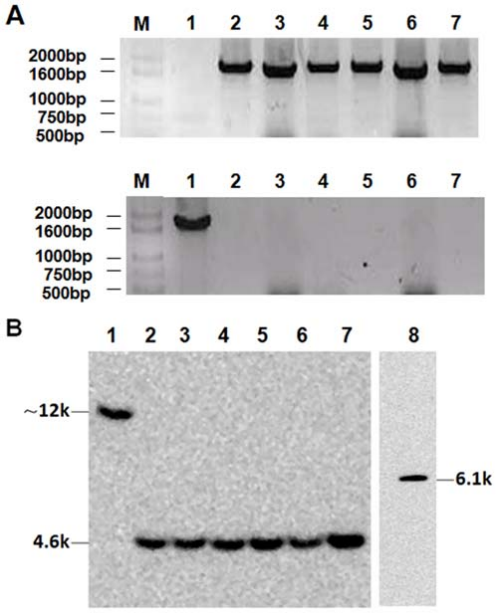
PCR and Southern analysis of the stability of the integrated locus in PfMSP1-19Pb8.7 clones. Genomic DNA was extracted from transgenic parasites at various times with wild type parasites as control. The primer pair of Tb5/Tf (upper line) was used to detect the integrated fragment in the transgenic parasite, while the primer pair of Tb5/T-Pb (lower line) was used to amplify the native sequence of PbMSP1 in the wild-type parasites used as controls (A). Ten micrograms of genomic DNA of wild type and transgenic *P. berghei* parasites collected at different times was digested with HincII and hybridized to probe PbM while the HincII-digested transfection plasmid served as positive control (B). M: base pair ladder; lane 1: the genomic DNA of wild type parasites; lanes 2–7: the genomic DNA of PfMSP1-19Pb_8.7_ clone that was extracted at various times (days 0,7,14,21,28 and 35); lane 8: the DNA of transfection plasmid digested by HincII.

The parasite clone 8.7 (termed PfMSP1-19Pb_8.7_) was selected for further Southern analysis and ‘passage stability’ assays. The duration of the developmental asexual blood-stage of *P. berghei* ANKA is 21 h. The parasite clone was allowed to proliferate in vivo without drug pressure for 35 days and collected at weekly intervals. Two PCR amplifications were performed. The 1.8-kb integrated MSP1 locus was amplified using the Tb5/Tf primers from all of the templates of transgenic parasites collected at the different times whereas a 1.8-kb fragment was amplified only from wild-type parasites using the Tb5/T-Pb primers (figures 2A). The same quantity (10 µg) of genomic DNA of wild type and transgenic *P. berghei* parasites was also digested with HincII restriction enzyme and hybridized to probe PbM, which is specific to the DNA sequences immediately upstream of *P. berghei* MSP1-19. Digestion of the genomic DNA with this restriction enzyme should generate a 12-kb fragment for the wild type *P. berghei* but a 4.6-kb fragment for the transgenic parasite that hybridized to this probe. As shown in figure 2B, the bands corresponding to the wild-type and transgenic *P. berghei* (i.e. 12 kb and 4.6 kb, respectively) were detected by Southern blotting. Moreover, the signal produced by the hybridization of the gene in the wild-type *P. berghei* is similar to that of the transgenic parasite, and also the signals are similar among the samples of the transgenic parasite collected from various time points during the successive passage.

### Green fluorescent detection and flow cytometry analysis of transgenic parasites in blood stages

**Figure 3:**
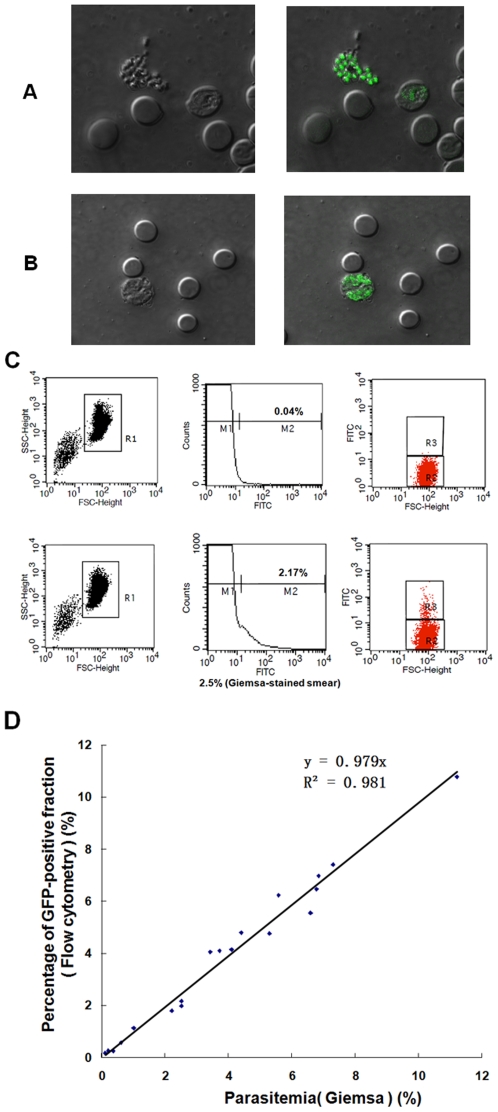
GFP expression and flow cytometry analysis of PfMSP1-19Pb_8.7_ clone in blood-stages. The same fields were photographed using bright (left panel) and fluorescence microscopy (right panel). Mature schizont stage (A). Early schizont stage (B). Flow cytometry analysis of blood cells obtained from infected mice with asynchronous infections. Per sample, 10^6^ erythrocytes were analyzed. Dot plots of blood cells were obtained from uninfected (upper line) and PfMSP1-19Pb_8.7_-infected (lower line) mice. For counting the number of infected cells the erythrocyte population was selected by size (forward-scatter (FSC) and side-scatter (SSC)) as shown in the left panel. The center panel shows the relative GFP-fluorescence intensity of cells in the R1 region. Fluorescent erythrocytes were selected from region M2. The right panel shows the dot plot of fluorescence intensity versus the size of cells in R1. Fluorescent erythrocytes were selected in R3, non-fluorescent in R2 (C). Correlation between the parasitemia counted by flow cytometry and the parasitemia counted manually in Giemsa stained blood films (D).

Blood was collected from the mice infected with transgenic clone 8.7 (PfMSP1-19Pb_8.7_), and cells were examined by fluorescence microscopy. Transgenic *P. berghei* in all erythrocyte stages displayed clear fluorescent signals (figures 3A and B).

To determine whether all of the transgenic parasites of the blood stages showed green fluorescence and could be readily differentiated from other blood cells using flow cytometry, blood samples were collected daily from PfMSP1-19Pb_8.7_-infected mice. For each sample, 10^6^ erythrocytes were analyzed for parasitemia using FACScalibur and Cell Quest software (figure 3C). Simultaneously, the parasitemia of each sample was also determined on Gimesa-stained smears. In these experiments, we observed a good agreement between the counting of infected cells by flow cytometry and manual counting of Giemsa-stained blood smears. A strong correlation was found using Pearson's correlation analysis (n = 20, R^2^ = 0.981, P<0.001). The linear relationship in the parasitemia from the two methods is shown in figure 3D.

### Phenotypic analysis of transgenic malaria parasites

**Figure 4:**
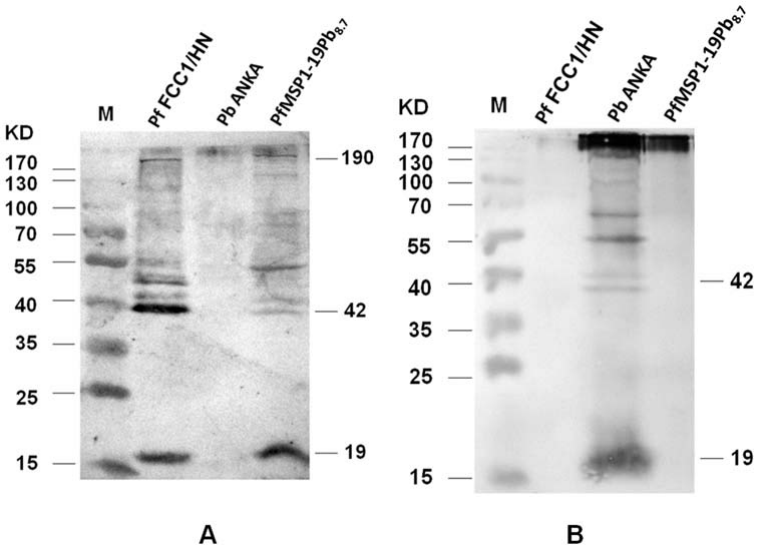
Western blot analysis of the PfMSP1-19 expression in PfMSP1-19Pb_8.7_ clone. Proteins extracted from enriched schizont preparations of parental *P. berghei* ANKA, transgenic PfMSP1-19Pb_8.7_ clone and *P. falciparum* FCC1/HN, respectively, were probed with either anti-PfMSP1-19 rabbit sera (A) or with anti-PbCP-2.9 mouse sera (B).

To determine whether the transgenic *P. berghei* clones expressed heterologous MSP1-19 domains from *P. falciparum*, Western blot analysis was performed on late-stage parasite extracts from *P. falciparum* FCC1/HN, wild-type *P. berghei* ANKA and the transgenic PfMSP1-19Pb_8.7_ clone. Purified rabbit antibodies to *P. falciparum* MSP1-19 recognized not only a major 19-kDa band but also the 42-kDa and full-length bands in *P. falciparum* FCC1/HN and PfMSP1-19Pb_8.7_, but did not interact with the corresponding molecules in the wild-type *P. berghei* ANKA (figure 4A). In contrast, the anti-PbCP-2.9 mouse sera only recognized the 19-kDa and 42-kDa bands in the wild-type *P. berghei* ANKA, but not in either *P. falciparum* FCC1/HN or PfMSP1-19Pb_8.7_ (figure 4B). These results demonstrated that PbfMSP-1 was expressed in transgenic parasites that were generated by replacing the corresponding domain in *P. berghei* by the PfMSP1-19.

**Figure 5:**
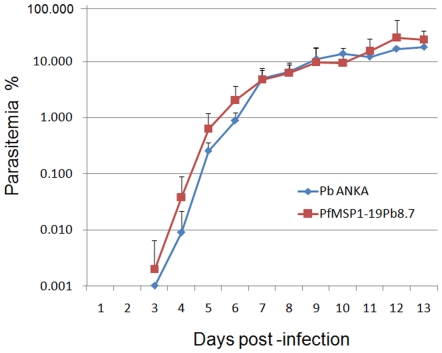
Comparison of parasitemia between the transgenic PfMSP1-19Pb_8.7_ parasite and its parental *P. berghei*. Two groups of mice (5 mice/group) were infected with 10^4^ erythrocytes harboring either wild-type *P. berghei* ANKA or PfMSP1-19Pb_8.7_ clone. The parasitemia was assessed daily using Giemsa-stained thin smears.

The growth rates of transgenic clones were also examined and compared to wild-type *P. berghei* parasite. Parasitemia was observed in both mouse groups over a similar time frame and showed no statistical differences (figure 5).

### The efficacy of passive transfer of the anti-PfCP-2.9 antibodies

A group of 3 rabbits was immunized with PfCP-2.9 emulsified in ISA720 adjuvant. After three immunizations, the reciprocal antibody titers for each immunized rabbit were 3,200,000, 1,200,000 and 850,000, respectively, as measured by ELISA. We chose the rabbit serum with the highest antibody titer to purify the total IgG that was used for passive transfer.

**Figure 6:**
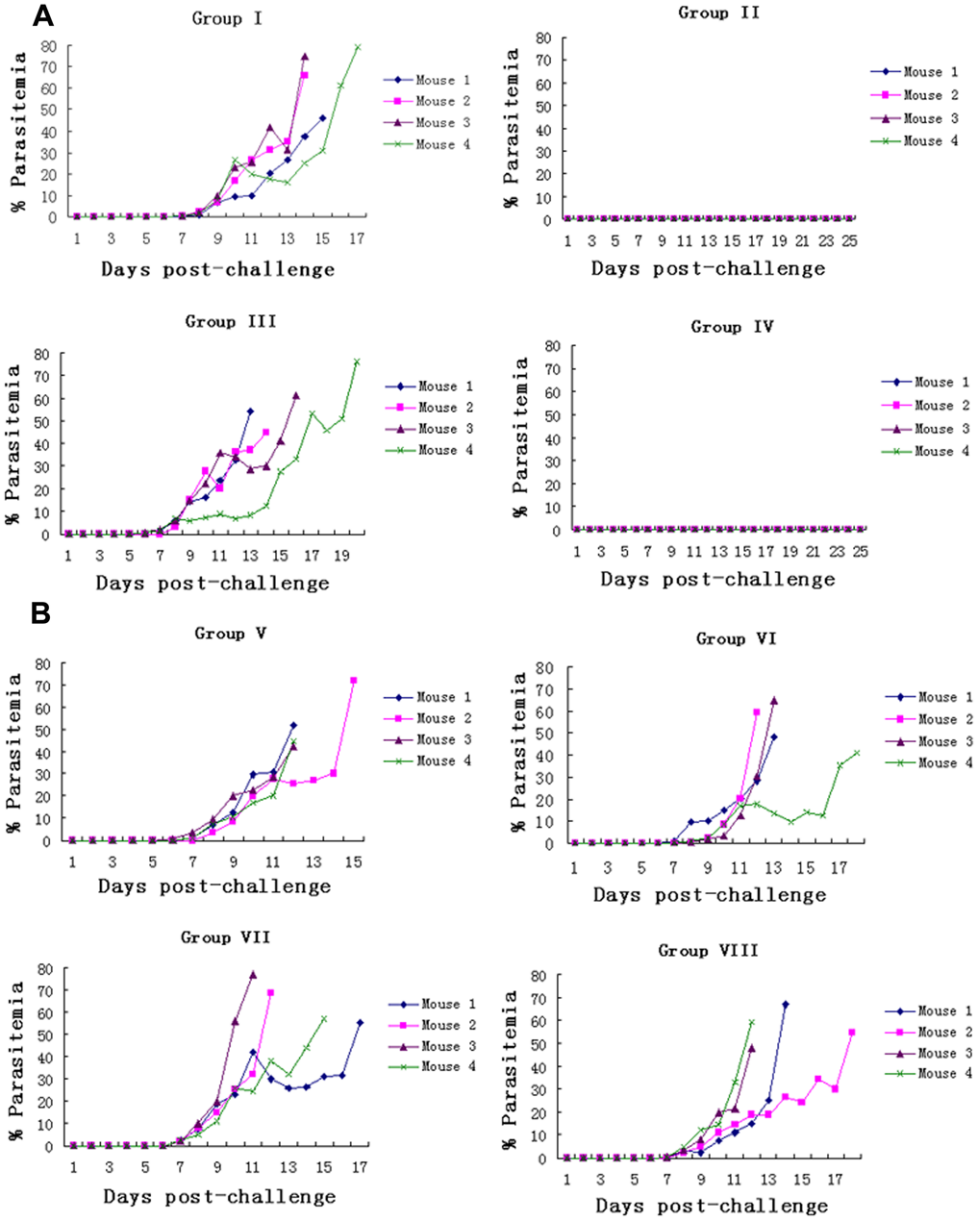
Parasitemia profiles of the mice in the passive transfer experiment. Groups of mice were injected with a total amount of 1.5 mg IgG (groups I and II) and 3.0 mg IgG (groups III and IV). (A): the mice in groups I and III were given IgG from adjuvant-only immunized rabbits, while mice in groups II and IV received IgG purified from rabbits immunized with PfCP-2.9 antigen. All mice were challenged with PfMSP1-19Pb_8.7_ transgenic parasite. (B): the mice were grouped and passively transferred with IgG in the same way as in (A), but were challenged with wild-type *P. berghei*.

To assess the protective efficacy of the anti-PfCP-2.9 antibodies, total IgG from PfCP-2.9-immmunized and adjuvant-immunized rabbits was passively transferred to BALB/c mice. The mice in groups I and III were intravenously given IgG from adjuvant-only treated animals (total doses of 1.5 and 3.0 mg, respectively) as negative controls while the mice in groups II and IV received anti-PfCP-2.9 IgG (total doses of 1.5 and 3.0 mg, respectively). Mice from all groups were challenged with transgenic *P. berghei*. Mice in the control group showed a continuous increase in parasitemia and died by day 21. Conversely, mice passively transferred with anti-PfCP-2.9 IgG survived, and no parasites were detected in the blood up to 25 days following the lethal challenge (figure 6A). To eliminate the possibility of non-specific protection or anti-PfCP-2.9 IgG cross-reactivity with *P. berghei* MSP1-19, four additional groups of mice (groups VI-VIII) were immunized in a similar fashion but challenged with wild-type *P. berghei*. All of the mice in these groups developed an infection and died by day 23 (figure 6B). These results suggested that antibodies to the PfCP-2.9 were protective only against the transgenic *P. berghei* parasites that contain the isoform of *Pf* MSP1-19.

## Discussion

Transfection and gene targeting of *Plasmodium* parasites can subtly disrupt, modify or replace genes, providing important genetic tools to better understand gene function of malaria parasites and their associated biological processes. To date, transfection systems have been successful in some *Plasmodium* species including the human, primate and rodent malaria parasites [32]–[35]. The fact that an MSP1 deletion was lethal indicated that this protein was essential for development of malaria parasites. However, allelic replacement of the MSP1-19 across distantly-related *Plasmodium* species has been successful [24], [36], [37], demonstrating that the function of the MSP1-19 fragment was fully conserved *in vivo* across species. The transgenic parasite lines have been utilized for biological studies of the malaria parasite including the in vivo evaluation of the inhibition function of specific antibodies [21], [22]. To evaluate the function of antibodies to the PfCP-2.9 that contains the MSP1-19, we used gene targeting in the *P. berghei* ANKA strain to construct a genetically modified murine parasite in which the MSP1-19 of *P. berghei* was replaced by the corresponding domain of *P. falciparum*. The resulting transgenic parasites were demonstrated to express the Pb/Pf MSP-1 chimeric protein. The growth characteristics of the transgenic parasites were similar to that of the wild type *P. berghei* parasites throughout the blood stages.

The green fluorescent protein (GFP) is a reporter protein that has been widely used because it can easily be measured in living organisms by fluorescence microscopy and flow cytometry. Therefore, the transgenic parasites were constructed to express GFP protein in addition to the PfMSP1-19. We found that the GFP-expressing transgenic parasites in all blood stages could be observed under a fluorescence microscope and that the parasitemia in the infected mice could be readily counted by flow cytometry. The advantages of the simultaneous expression of the Pb/Pf MSP-1 chimeric protein and GFP protein could be easy detection of transgenic parasites during the blood stage and the automated counting of parasitemia, which can potentially be used for determination of in vivo vaccine efficacy and other biological studies of malaria parasites related to MSP-1. In addition, with the transgenic parasites, strain-specific protection can be analyzed in mice against a challenge of mixed wild-type *P. berghei* and transgenic parasites. Although other studies have generated transgenic parasites that express either PfMSP1-19 or GFP protein, the simultaneous expression of the PfMSP1-19 and GFP should be superior to the expression of the molecules separately.

To create the transgenic *P. berghei* parasites, a pPyrFlu plasmid that can express a pyrimethamine resistant gene and a GFP gene as a DHFR-TS/GFP fusion protein was used [28]. Under the control of the P. berghei DHFR-TS 5′UTR, the fusion protein could be constitutively expressed during the asexual RBC phase. Since pyrimethamine selection frequently yields a proportion of spontaneously resistant mutants that cannot be eliminated even with higher drug doses and longer selection times, the selected *P. berghei* parasite population actually contained both wild-type and transgenic parasites. To identify the transgenic parasite clones, the pyrimethamine-resistant parasites underwent two rounds of cloning using the limiting dilution method. The parasite clones were firstly verified by PCR analysis using five primer sets to determine whether the integration and replacement occurred correctly. As a result, only four resistant clones showed the desired integration that was further confirmed by Southern blot analysis. The stability of the integrated MSP-1 locus was also shown by Southern analysis during 35 days of successive passages, demonstrating that no reversion to the wild type had occurred. To determine the phenotype of the transgenic *P. berghei* parasites, one of the identified clones was chosen for analysis. The results of Western blot analysis confirmed that only *P. falciparum* MSP1-19 was expressed in transgenic murine parasites, while the expression of the endogenous PbMSP1-19 gene was not detected. A comparison of amino acid sequence of PfMSP1-19 with that of PbMSP1-19 indicates only 35% sequence identity and the lack of one disulfide bond in the first EGF domain in *P. berghei*. However, the detection of the full-length and processed forms of chimeric PbfMSP-1 protein indicates that the proteolytic processing occurs correctly in the transgenic *P. berghei* parasite and releases the heterogenous PfMSP1-19. In addition, the growth rate of transgenic *P. berghei* showed no statistical differences compared to that of the wild-type. Our results demonstrated that the replacement of the MSP1-19 region and the expression of DHFR-TS/GFP fusion protein were not deleterious to the transgenic *P. berghei* parasites. These results were consistent with previous findings for functional conservation of MSP1-19 domain between human and murine *Plasmodium* species [24], [36], [37].

Humans are the natural host for *P. falciparum*, and the lack of animal models for infections with this *Plasmodium* species makes the discovery of vaccine candidates challenging. The evaluation of the immune protective efficacies of malaria vaccines often involves the use of robust and reliable assays. The *in vitro* Growth Inhibition Assay is a rapid and inexpensive way of evaluating protective immunity and provides valuable information on the function of immune sera, but an accurate and full assessment of inhibitory activity with this *in vitro* method is difficult. Moreover, at present there is no demonstrable *in vitro* assay that directly correlates protective immune efficacy. In this study, we generated a transgenic *P. berghei* that expresses both *P. falciparum* MSP1-19 and GFP protein simultaneously, providing a model to further test the protective efficacy of the PfCP-2.9 vaccine. We found that only mice given the purified IgG from PfCP-2.9-vaccinated rabbits survived a lethal challenge of transgenic murine parasites, indicating that PfCP-2.9 elicited the production of inhibitory anti-MSP1-19 antibodies that provided protection against the parasite *in vivo* at a low dose parasite challenge (500 infected RBC per mouse). The fact that all of the mice that were also given IgG from PfCP-2.9-vaccinated rabbits developed an infection of wild-type *P. berghei* eliminated the possible cross-species protection of antibodies elicited by PfCP-2.9. Taken together, using gene targeting we generated a *P. berghei* line that expresses PfMSP1-19 and the GFP reporter gene simultaneously. In this transgenic line, MSP1-19 derived from *P. falciparum* complemented the *in vivo* function of its own MSP1-19 domain, at least in the erythrocytic stage, although *P. berghei* MSP1-19 lacks one disulfide bond in the first EGF domain and shares only 35% amino acid identity to *P. falciparum* MSP1-19. Transgenic parasites are similar to the wild-type parasites in proliferation and invasion of erythrocytes. The availability of transgenic *P. berghei* that expresses *P. falciparum* MSP1-19 and GFP protein provides a murine model to evaluate protective efficacy in vivo of anti-MSP1-19 antibodies, including those elicited by the PfCP-2.9 malaria vaccine in human volunteers.
